# Adapting Classification Neural Network Architectures for Medical Image Segmentation Using Explainable AI

**DOI:** 10.3390/jimaging11020055

**Published:** 2025-02-13

**Authors:** Arturs Nikulins, Edgars Edelmers, Kaspars Sudars, Inese Polaka

**Affiliations:** 1 Faculty of Computer Science, Information Technology and Energy, Riga Technical University, LV-1048 Riga, Latvia; inese.polaka@rtu.lv; 2 Faculty of Medicine, Rīga Stradiņš University, LV-1010 Riga, Latvia; 3 Institute of Electronics and Computer Science, LV-1006 Riga, Latvia; sudars@edi.lv

**Keywords:** medical imaging, classification models, image segmentation, explainable artificial intelligence, neural networks

## Abstract

Segmentation neural networks are widely used in medical imaging to identify anomalies that may impact patient health. Despite their effectiveness, these networks face significant challenges, including the need for extensive annotated patient data, time-consuming manual segmentation processes and restricted data access due to privacy concerns. In contrast, classification neural networks, similar to segmentation neural networks, capture essential parameters for identifying objects during training. This paper leverages this characteristic, combined with explainable artificial intelligence (XAI) techniques, to address the challenges of segmentation. By adapting classification neural networks for segmentation tasks, the proposed approach reduces dependency on manual segmentation. To demonstrate this concept, the Medical Segmentation Decathlon ‘Brain Tumours’ dataset was utilised. A ResNet classification neural network was trained, and XAI tools were applied to generate segmentation-like outputs. Our findings reveal that GuidedBackprop is among the most efficient and effective methods, producing heatmaps that closely resemble segmentation masks by accurately highlighting the entirety of the target object.

## 1. Introduction

Before integrating AI predictions into medical decision making, it is important to ensure their safety and accuracy. Explainable artificial intelligence (XAI) tools are commonly used to achieve this goal. XAI tools reveal where the model focuses its attention when making predictions. However, can these attention maps be effectively used for segmentation tasks? Exploring AI applications is valuable for uncovering intricate details within diverse medical imaging datasets, including magnetic resonance imaging (MRI), Computed Tomography (CT), ultrasonography and others [1,2,3,4]. For example, segmentation can offer several advantages over other AI methods, especially for radiology data analyses. Such advantages include clear indication of where issues can occur, clear identification of boundaries between healthy and unhealthy tissue and also quantitative analysis enabling the precise measurement of tumour sizes, lesion volumes or other pathological changes.

However, the neural network data classification approach is faster and does not require extensive and pressed manual data annotation, as is necessary in the segmentation approach. The primary distinction between segmentation and classification network architectures lies in the presence of a decoder. Classification architectures are designed to compress input data into a latent space, ultimately producing a compact representation, such as a class label. In contrast, segmentation architectures encode the input image into a latent space and then decode this representation back into a pixel-wise, human-interpretable form. From this perspective, explainable AI (XAI) tools can be viewed as functioning similarly to decoders, as they extract and translate encoded latent space information into an interpretable format.

It is worth mentioning that XAI tools are designed to provide transparency and interpretability in AI models. Their purpose is to offer insights into decision-making processes, making it easier for users to understand why a particular prediction or classification was made. We propose using these explanation-generated masks as substitutes for traditional segmentation masks.

Many open-access competitive projects that incorporate XAI are available [5,6,7,8,9,10]. The aim of these tools is to analyse both glass-box and black-box systems, such as neural networks, to ensure their proper functioning. In this paper, we compare different XAI libraries available in the Python environment for analysing classification neural networks. A comparison of libraries and methods can help identify the most effective ones for classification tasks in medical imaging.

We chose Captum among the Python libraries for XAI tool exploration and compared the most commonly used tools available in the Captum library with different analysis methods. The tools in the Captum library feature diverse working methods and offer different types of analysis, such as single-neuron analysis, the analysis of one layer of the neural network and input image analysis. Each tool brings a unique approach to understanding the operation of AI models. We engage in a discussion to determine which tool in the Captum library provides the most precise information for our objectives. From the Captum library for computer vision tasks, some of the most popular methods include Integrated Gradients, Gradient SHAP, Guided Backpropagation and Occlusion [11,12,13,14]. Each of these methods offers a unique approach to interpret and explain the decisions made by neural network models in visual data processing. These methods utilise various approaches, including gradient-based methods, perturbation-based methods, layer attribute and Neuron Attribution methods. By exploiting these methods, we believe that it is possible to mark a region where the desired class is located, therefore adapting the classification neural network for the segmentation task.

The motivation for this paper stems from studies of segmentation and classification neural networks, their latent spaces and XAI. We identified similarities in how both types of networks process and represent data, particularly in the latent space, where important object parameters are encoded. These observations inspired us to explore and compare XAI-generated masks with traditional segmentation masks, especially in the medical domain, where segmentation networks are widely used but face significant challenges and restrictions.

This study introduces a novel approach to adapting classification neural networks for medical image segmentation by leveraging explainable AI (XAI) tools, providing an alternative to traditional segmentation networks that require extensive manual annotations and specialised architectures. By utilising XAI methods such as GuidedBackprop and Grad-CAM, the proposed methodology generates segmentation-like masks from classification models, serving as surrogate decoders to bridge the gap between classification and segmentation tasks. The approach reduces dependency on detailed pixel-wise annotations, making it particularly valuable for medical imaging applications where annotated data are scarce or restricted. Although demonstrated on the ‘Brain Tumours’ dataset, this method is broadly applicable to other domains, including histopathology and radiology. A robust postprocessing pipeline further refines XAI-generated heatmaps into accurate binary segmentation masks, addressing issues like noise and incomplete delineation. By exploring the potential of repurposing classification networks for segmentation, this study fills a gap in the literature and establishes a foundation for future research on adapting classification models for segmentation tasks across diverse applications.

This publication is constructed as follows: In the Methodology section, we define the dataset used for our experiments and describe the neural network architecture employed. We then explore and compare various XAI Python libraries from a theoretical perspective, ultimately selecting Captum as the most suitable library for our needs. Following this, we delve into the exploration of different XAI methods available in Captum. The Results section presents the performance of the trained classification neural network and demonstrates how XAI methods successfully generate segmentation-like masks. In the Discussion section, we evaluate the best XAI methods for this task, considering both processing time and mask precision. Finally, we conclude with a summary of our findings and their implications for future work.

## 2. Materials and Methods

Adapting a classification system to perform segmentation tasks is challenging but achievable. We used the ‘Brain Tumours’ database, which is available on the official Medical Segmentation Decathlon website, for our data needs. The ‘Brain Tumours’ database consists of the BraTS 2016 and 2017 datasets. In BraTS 2016, the training set included 220 high-grade glioma (HGG) and 54 low-grade glioma (LGG) cases, while the testing set had 191 cases with unspecified grades. For BraTS 2017, the training set consisted of 210 HGG and 75 LGG cases; the validation set contained 46 cases, and the testing set had 146 cases, with grades not specified for the latter two sets [15]. The dataset was stored in an NIfTI (.nii.gz) container, commonly used for 3D medical volumes. We transformed the 3D volumes into multiple 2D slices, each representing an axial cut of the human brain. Each image is 240 × 240 pixels in size and has three (RGB) channels. For analyses, the data were transformed into 512 × 512 pixels. This transformation was applied for specific architecture input size. The dataset includes tumours that are most clearly visible in the second imaging modality.

For our experiment, we trained the smallest architecture of the ResNet model in two distinct cases: slices with tumour and slices without tumour. The main goal is to use XAI to identify the precise location of the tumour in the image. The focus should be solely on the tumour, excluding any surrounding head or healthy tissues lines.

We modified the ResNet-18 architecture by changing the last layer to output two classes, as our specific task involves binary classification rather than the original 1000 classes for which ResNet-18 is designed. It is important to note that no other changes were made to the ResNet-18 architecture to adapt it for segmentation. Essentially, the ResNet-18 architecture for classification task functions similarly to the encoder part of a segmentation neural network and creates latent space at the end, while the XAI tools emulate the functionality of the decoder part by generating masks that utilise and take into account the important object parameters encoded within the latent space.

### 2.1. XAI Library Choice

We compared different available projects that encapsulate the XAI tools. InterpretML, developed by Microsoft, supports a range of techniques for both glass-box and black-box models. For glass-box models, it includes Explainable Boosting, APLR, Decision Tree, Decision Rule List and Linear/Logistic Regression. For black-box models, it supports SHAP Kernel Explainer, LIME, Morris Sensitivity Analysis and Partial Dependence [7]. Currently, the InterpretML library is primarily geared towards tabular data and does not natively support image data or computer vision tasks. In contrast, Alibi offers a variety of techniques for image and computer vision analyses, including Anchors, CEM, and Counterfactuals [8]. Alibi supports both global and local interpretability methods. It includes state-of-the-art explainability techniques and provides two evaluation metrics. AIX360, developed by IBM, supports LIME and SHAP, which are among the most popular algorithms in the XAI field. In addition to these methods, AIX360 offers a comprehensive toolkit for interpretability and fairness in machine learning models [9]. Many other reviewed projects, which are not mentioned, have various limitations, such as a lack of support for PyTorch models, the inability to handle computer vision tasks and the inability to support many state-of-the-art algorithms. At the end of our theoretical research, we chose the Captum library, as it stands out due to its comprehensive support for PyTorch models and computer vision tasks. It consolidates all the best tools available and excels at providing state-of-the-art methods that offer detailed explanations. Captum allows for the analysis of individual neurons, hidden layers and data through comprehensive analyses of the entire architecture. It supports a wide range of algorithms, including LIME, SHAP and Grad-CAM, making it a versatile tool for model interpretability [5].

The classification network was trained on an NVIDIA A100 GPU, while the XAI heatmaps were generated by using an NVIDIA GeForce GTX 1050 Ti GPU (4 GB GPU memory) and an Intel Core i5-8400 CPU at 2.80 GHz. Generating XAI heatmaps can be performed on an average computer system; however, sufficient GPU memory is required for XAI methods. In our experiments, the system was equipped with 4 GB of GPU memory. Backpropagation-based tools, such as Guided Grad-CAM and Guided Backpropagation, do not demand high GPU memory, whereas occlusion-based methods often require more than 4 GB of GPU memory.

### 2.2. Captum

We reviewed tools employing various methods and selected the best ones suited for our classification task. Specifically, we focus on tools that provide clear and interpretable insights into the neural network decision-making process. These tools were then applied to the trained ResNet-18 classification model to enhance its explainability and ensure that the results are accurate and understandable. Captum offers different methods to achieve explainability. For example, the Occlusion method systematically occludes parts of the input to identify the most important regions for the neural network’s prediction; saliency calculates the gradients of the output with respect to the input, highlighting the areas that have the most influence on the network’s decision [10]; Guided Grad-CAM combines gradient information and feature maps to produce a localisation map, showing which parts of the input contribute most significantly to the decision-making process [6], and many other XAI tools which are available using Captum provide deeper insights into the inner workings of models. In the Captum library, all algorithms are standardised in their use. To activate a single algorithm using Captum, it is only necessary to call two functions: create an instance of the specific tool and activate the attributes of this specific tool. In the computer vision task, every algorithm gives representations using heatmaps to indicate where AI is focussing its attention for prediction.

Our classification task involved training ResNet-18 on two classes. All XAI algorithms or tools in Captum can be classified into three categories: tools that analyse a single neuron, tools that analyse a single hidden layer and tools that analyse the entire model by comparing input with output.

Tools that analyse a single neuron focus on understanding the behaviour and influence of individual neurons for output prediction within the network. For example, tools like NeuronConductance [16] and NeuronGradient. Those that analyse a single hidden layer provide insights into the role and function of specific layers, revealing how they contribute to the overall decision-making process, for example, LayerGradientShap [12], LayerGradientXActivation [17] and LayerIntegratedGradients [11,17]. Lastly, tools that analyse the entire model compare the input and output to offer a comprehensive view of the network’s performance and decision-making patterns, highlighting which aspects of the input are the most significant for the model’s predictions, for example, FeatureAblation, GuidedBackprop [18] and GuidedGradCam [6]. We utilised all these tools to gain a deeper understanding of the model’s workings and to identify the most effective methods for simulating segmentation neural network results.

One of the notable features of the Captum library is the ability to process single-channel images. However, a tool like LIME outside the Captum library specifically requires three-channel RGB images and will issue an alert indicating that only RGB images are compatible for processing [16]. The Captum library’s ability to analyse single-channel images is especially advantageous for neural networks used in medical tasks, considering that standard medical imaging techniques such as CT, MRI and ultrasonography usually rely on one-channel representation.

### 2.3. Enhancing Segmentation Accuracy Through Postprocessing

Postprocessing plays an important role in generating the final binary mask. After constructing the heatmap, a threshold is applied to identify significant regions associated with the tumour class. This process often results in outlined regions rather than filled areas. To achieve a complete and accurate segmentation mask, additional steps like filling enclosed regions are required, as depicted in Algorithm 1.
**Algorithm 1:** Enhancing segmentation accuracy through postprocessingdef fill_area(image_shape, points):  image = np.zeros(image_shape, dtype=np.uint8)  points = np.flip(points, axis=1)  cv2.fillPoly(image, [points], 255)  return image def process_mask(image_path):  image = mpimg.imread(image_path)  if image.shape[-1] == 4:   image = image[..., :3]  image = rgb2gray(image)  image[image <= 0.35] = 0  image[image != 0] = 1  output_img = process_clusters(image)  closed_image = analyze_and_fill_clusters(output_img, kernel_size=5)  coords = np.column_stack(np.where(image > 0))  if len(coords) >= 3:   hull = ConvexHull(coords)   hull_coords = coords[hull.vertices]   filled_image = fill_area(image.shape, hull_coords)   return filled_image  else:   return np.zeros(image.shape, dtype=np.uint8) def analyze_and_fill_clusters(image, kernel_size=5):  filled_image = binary_fill_holes(image).astype(np.uint8)  kernel = cv2.getStructuringElement(cv2.MORPH_ELLIPSE, (kernel_size, kernel_size))  closed_image = cv2.morphologyEx(filled_image, cv2.MORPH_CLOSE, kernel)  return closed_image def process_clusters(image):  labeled_image, num_clusters = label(image)  output_image = np.zeros_like(image, dtype=np.float32)  for cluster_id in range(1, num_clusters + 1):   cluster = (labeled_image == cluster_id).astype(np.uint8)   cluster_size = np.sum(cluster)   if cluster_size < 10:    continue   else:    padded_image = np.pad(cluster, pad_width=2, mode='constant', constant_values=0)    kernel = np.ones((5, 5), dtype=np.uint8)    neighbors = cv2.filter2D(padded_image, -1, kernel)    neighbors = neighbors[2:-2, 2:-2]     if np.sum(neighbors) > np.sum(cluster):     expanded_cluster = cv2.dilate(cluster, kernel=np.ones((3, 3), dtype=np.uint8))     output_image = np.maximum(output_image, expanded_cluster)  return output_image mask_path = "results/GuidedBackprop.png" mask_filled = process_mask(mask_path) original_image = mpimg.imread("Brain_decathlon/processed_slices/tumor/BRATS_007_slice_107_modality_2.png") if original_image.shape[-1] == 4:  original_image = original_image[..., :3] mask_filled_resized = cv2.resize(mask_filled, (original_image.shape[1], original_image.shape[0])) mask_rgb = cv2.cvtColor(mask_filled_resized, cv2.COLOR_GRAY2RGB) if original_image.dtype != np.uint8:  original_image = (original_image * 255).astype(np.uint8) mask_filled_resized = cv2.resize(mask_filled, (original_image.shape[1], original_image.shape[0])) mask_rgb = cv2.cvtColor(mask_filled_resized, cv2.COLOR_GRAY2RGB) overlay = cv2.addWeighted(original_image, 1.0, mask_rgb, 0.7, 0)

## 3. Results

During the training process of the classification ResNet model, the training and validation accuracy rates reached 100% and 99.69%, respectively, at epoch 200, where the model achieved convergence. The corresponding training and validation losses were 0.001 and 0.008. It is important for the tumour to be well defined in order to generate accurate XAI masks.

If the tumour is well defined, the neural network focuses only on parameters within the tumour class, disregarding irrelevant features such as brain structures or head morphology.

**Figure 1:**
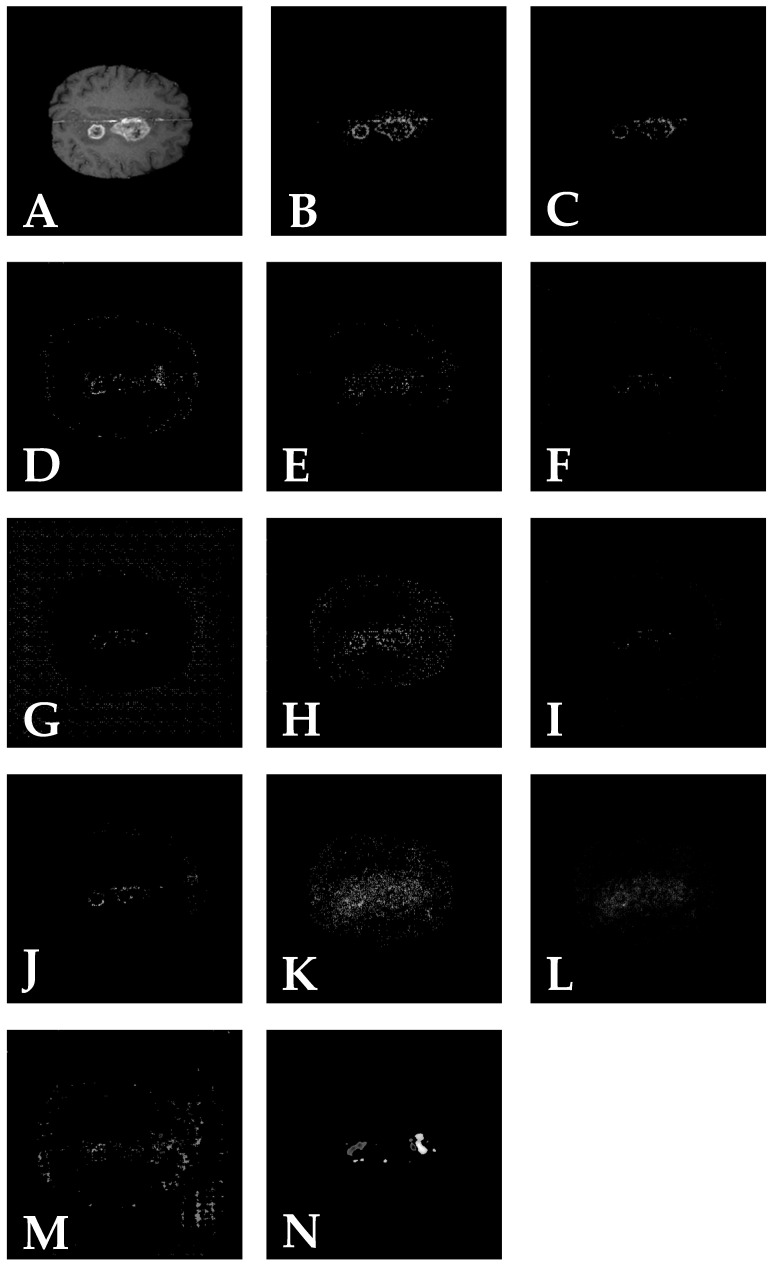
Original image (**A**), GuidedBackprop (**B**), GuidedGradCam (**C**), InputXGradient (**D**), InternalInfluence (**E**), LayerConductance (**F**), LayerGradientShap (**G**), LayerGradientXActivation (**H**), LayerIntegratedGradients (**I**), NeuronConductance (**J**), NeuronGradient (**K**), Saliency (**L**), FeatureAblation (**M**) and Occlusion (**N**).

Different tools within Captum library have a different way to analyse a model; therefore they have different interpretation of the model. The Occlusion method provides a clear representation of the model focus point, which is the tumour itself, as illustrated in Figure 1N. Other tools provide decent explanation results, mainly represented as point clouds concentrated near the tumour region. It is important to note that tools such as NeuronConductance (Figure 1J) and NeuronGradient (Figure 1K) were specifically designed to analyse a single neuron within the neural network. Meanwhile, tools such as LayerConductance (Figure 1F), LayerGradientShap (Figure 1G), LayerGradientXActivation (Figure 1H) and LayerIntegratedGradients (Figure 1I) are used to analyse a specific hidden layer within the neural network.

Tools based on individual neural network layer or neuron analysis methods (LayerConductance, LayerGradientShap, NeuronConductance, etc.) can produce multiple channels after analysis. For example, the LayerConductance method, when analysing the first convolutional layer of the ResNet-18 architecture, generates an output with dimensions (1, 64, 112, 112) from an input image with dimensions (1, 1, 224, 224). Only 1 of the 64 channels is represented as the LayerConductance output result (Figure 1F), but in practice, 64 resulting images are available. The depicted image best represents the model’s focus on the tumour. The specific channel was selected manually. This is a limitation for layer-based and neuron-based methods of heatmap generation, as each mask must be analysed manually to choose the most suitable one.

The GuidedGradCam and GuidedBackprop methods provide a clear representation of tumours in images. Additionally, different filters can be applied during image analysis to adjust edge representation, reduce noise and fill the interior of shapes. An important observation is that all tools, except Occlusion and FeatureAblation, require less than a second to calculate attributions, demonstrating their efficiency in processing. GuidedBackprop provides a clear representation with minimum noise in the image, and the calculation time is less than a second; therefore, GuidedBackprop is a candidate tool for the most efficient performance in these kinds of tasks due to its ability to semi-mirror the shape of the tumour. Additionally, its processing time was 0.357 s. The calculation time was measured by using Python’s ‘time’ library. The time measurement involved recording the start time before applying the XAI method and the end time after the method completed the task.

The three examined tools provide the best representation of neural network operations. The GuidedBackprop method analyses the entire neural network as a unified system. Neuron analysis methods such as NeuronConductance focus on the analysis of a specific neuron, and layer methods such as LayerGradientShap focus on the analysis of a specific hidden layer. In practice, these tools are used to explain the functioning of a particular model. However, as can be seen, when applied to a classification neural network, some of these methods can also indicate the location of a specific class in an image, possibly repeating segmentation models’ performance. As can be seen, the most promising method that could theoretically repeat segmentation models’ performance are those that analyse input data based on output results, effectively evaluating the entire model as a whole. In the second experiment, we selected GuidedGradCam from all input analysis methods due to its ability to semi-mirror the shape of the tumour.

The following table represents the total processing time for all methods (Table 1).

Postprocessing is important part as it allows for the creation of an actual binary mask. After constructing the heatmap, a threshold is applied to extract the significant regions corresponding to the tumour class. A common outcome of this process is the appearance of outlines rather than filled regions. Therefore, additional steps, such as filling the enclosed areas, are necessary to achieve a complete and accurate representation of the segmentation mask.

**Figure 2:**
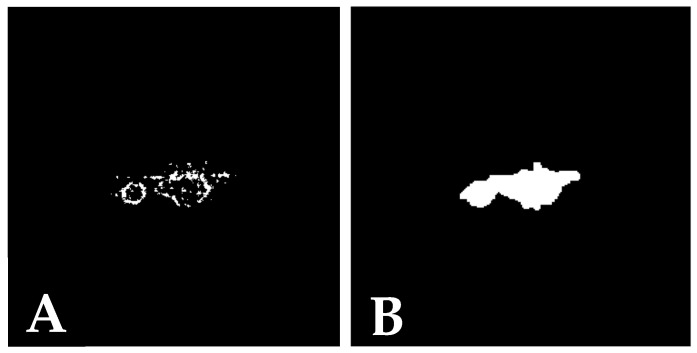
GuidedBackprop method prior postprocessing (**A**) and after postprocessing (**B**).

Binary thresholding, convex hull, polygon filling, clusters, binary fill holes and morphological transformations were applied in the postprocessing step to improve the segmentation mask. In the future, methods like these, along with other postprocessing techniques, can be further explored and refined to enhance segmentation accuracy and quality. The result of postprocessing is depicted in Figure 2.

**Figure 3:**
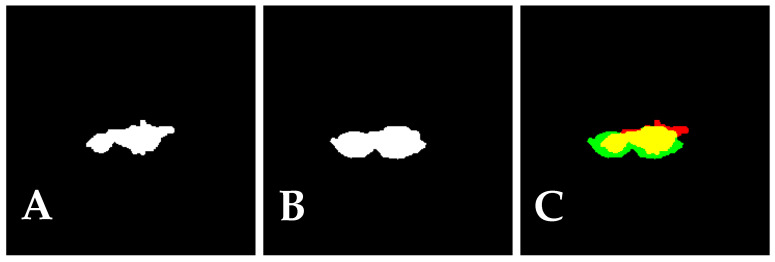
Metrics calculations. Predicted mask (**A**), ground-truth mask (**B**) and overlay of the two previous masks (**C**).

To evaluate mask accuracy, we consider semantic segmentation evaluation metrics. In the example depicted in Figure 3, the Dice score was 0.7319, and the IoU was 0.5771.

## 4. Discussion

Segmentation neural networks are valuable in medicine, particularly for analysing large datasets to identify problematic regions with precision. Recent advancements in this field are highlighted by significant publications, including the Segment Anything model for medical data, along with architectures such as EffiSegNet, DuckNet and many others. These architectures showcase diverse approaches and compete across various segmentation tasks in medical imaging, each aiming to improve performance and efficiency. The sheer size and complexity of medical datasets often make it difficult to manually locate regions of concern. Segmentation neural networks empower artificial intelligence to address this challenge by pinpointing critical areas, supporting early diagnosis and treatment planning. Given the unique demands of medical applications, segmentation remains a central focus in AI research and development [19,20,21,22].

Classification neural networks are useful for performing rapid tasks, such as fast classification into categories. Such categorisation can be beneficial, for example, for statistical purposes. This study demonstrates that it is also possible to adapt classification models for segmentation tasks within the medical realm by leveraging explainable AI techniques. The key contribution of this work lies in establishing a foundation for the idea of extracting important parameters from image objects encoded in the latent space of classification models to construct segmentation-like masks. This approach reduces the reliance on extensive manual segmentation while enabling the reuse of classification models for dual purposes, bridging the gap between classification and segmentation tasks. Additionally, this study highlights the practical application of XAI tools as surrogate decoders.

Despite its potential, this approach has several limitations. One challenge is that the boundaries of objects in medical images are often not well defined and may appear blurred, making it difficult for XAI-generated masks to precisely delineate the regions of interest. Additionally, while classification neural networks primarily focus on the object itself for identification, the surrounding context or specific regions of the object may carry information for accurate classification. This can lead to XAI-generated masks showing focus on the surrounding anatomical context rather than strictly on the object. Moreover, the application of explainability methods can require more processing time compared with dedicated segmentation neural networks. However, classification neural networks can offset this limitation by reducing the overall time required, particularly when factoring in the significant time and effort needed to manually annotate data for segmentation models. Many of these limitations could potentially be mitigated through postprocessing techniques, such as refining the boundaries or improving the clarity of the segmented regions. Techniques like morphological operations, conditional random fields and other refinement methods (e.g., binary thresholding, convex hull [23,24], polygon filling, clusters, binary fill holes and morphological transformations [25,26]) could help enhance the precision of the segmentation output. In future studies, we aim to improve postprocessing techniques to better resemble segmentation masks.

These adaptations of classification models are useful in cases where a classification model is trained on specific data that would be challenging to gather the second time for segmentation network training. As mentioned, training a segmentation network requires extensive time for labelling, and obtaining medical data is often difficult due to data protection regulations. An example of this is rare disease detection, where acquiring a large dataset of segmented images is nearly impossible due to the limited number of diagnosed cases and the high cost of expert annotations. In such scenarios, a classification model trained on available labelled data (e.g., where disease is present or not) can still be adapted for segmentation tasks by using explainability tools like GuidedBackprop or Grad-CAM to highlight regions of interest, bypassing the need for detailed segmentation labels. These limitations require improvisation in some cases, and one way to address this is to adapt a classification neural network for segmentation tasks.

GuidedBackprop is a strong candidate for achieving optimal performance in tasks like these due to its speed and clear representation of object location, making it an efficient tool for identifying areas of interest.

## 5. Conclusions

In this study, we showed that classification neural networks, combined with explainable AI tools, can generate segmentation-like outputs for medical images. By using methods like GuidedBackprop and GuidedGradCam, we found that it is possible to pinpoint a tumour region in an image, even though the model was trained only to classify images rather than to segment them. These methods work quickly—often in under a second—and provide clear maps that highlight the tumour with minimal extra noise. Postprocessing steps can then refine these maps into binary masks, improving accuracy. While this is a promising direction, it is not meant to replace well-established segmentation models, especially for complex or subtle medical findings. Instead, it serves as an alternative or supplement when detailed annotated data are limited.

## Figures and Tables

**Table 1:** Comparison of methods in processing time for mask creation.

Method	Processing Time in Seconds
InputXGradient	0.012
LayerGradientXActivation	0.012
NeuronGradient	0.012
LayerConductance	0.046
GuidedGradCam	0.084
NeuronConductance	0.192
LayerIntegratedGradients	0.201
Saliency	0.205
GuidedBackprop	0.357
LayerGradientShap	0.363
InternalInfluence	0.668
Occlusion	969.446
FeatureAblation	4253.165

## Data Availability

MedMNIST: https://zenodo.org/records/10519652 (accessed on 10 February 2025).

## References

[1] Shimron E., Perlman O. (2023). AI in MRI: Computational Frameworks for a Faster, Optimized, and Automated Imaging Workflow. Bioengineering.

[2] Bumm R., Zaffino P., Lasso A., Estépar R.S.J., Pieper S., Wasserthal J., Spadea M.F., Latshang T., Kawel-Boehm N., Wäckerlin A. (2024). Artificial Intelligence (AI)-Assisted Chest Computer Tomography (CT) Insights: A Study on Intensive Care Unit (ICU) Admittance Trends in 78 Coronavirus Disease 2019 (COVID-19) Patients. J. Thorac. Dis..

[3] Dicle O. (2023). Artificial Intelligence in Diagnostic Ultrasonography. Diagn. Interv. Radiol..

[4] Azeez I.I., Chow L.S., Solihin M.I., Ang C.K. (2023). 3D Brain Tumour Segmentation Using UNet with Quantitative Analysis of the Tumour Features. J. Phys. Conf. Ser..

[5] Kokhlikyan N., Miglani V., Martin M., Wang E., Alsallakh B., Reynolds J., Melnikov A., Kliushkina N., Araya C., Yan S. (2020). Captum: A Unified and Generic Model Interpretability Library for PyTorch 2020. arXiv.

[6] Selvaraju R.R., Cogswell M., Das A., Vedantam R., Parikh D., Batra D. (2020). Grad-CAM: Visual Explanations from Deep Networks via Gradient-Based Localization. Int. J. Comput. Vis..

[7] Nori H., Jenkins S., Koch P., Caruana R. (2019). InterpretML: A Unified Framework for Machine Learning Interpretability. arXiv.

[8] Klaise J., Looveren A.V., Vacanti G., Coca A. (2021). Alibi Explain: Algorithms for Explaining Machine Learning Models. J. Mach. Learn. Res..

[9] Arya V., Bellamy R.K.E., Chen P.-Y., Dhurandhar A., Hind M., Hoffman S.C., Houde S., Liao Q.V., Luss R., Mojsilović A. (2019). One Explanation Does Not Fit All: A Toolkit and Taxonomy of AI Explainability Techniques. arXiv.

[10] Simonyan K., Vedaldi A., Zisserman A. (2014). Deep Inside Convolutional Networks: Visualising Image Classification Models and Saliency Maps. arXiv.

[11] Sundararajan M., Taly A., Yan Q. (2017). Axiomatic Attribution for Deep Networks. https://proceedings.mlr.press/v70/sundararajan17a.html.

[12] Lundberg S., Lee S.-I. (2017). A Unified Approach to Interpreting Model Predictions. https://proceedings.neurips.cc/paper/2017/hash/8a20a8621978632d76c43dfd28b67767-Abstract.html.

[13] Merrick L. (2019). Randomized Ablation Feature Importance. arXiv.

[14] Zeiler M.D., Fergus R. (2014). Visualizing and Understanding Convolutional Networks. Computer Vision—ECCV 2014, Proceedings of the 13th European Conference, Zurich, Switzerland, 6–12 September 2014.

[15] Antonelli M., Reinke A., Bakas S., Farahani K., Kopp-Schneider A., Landman B.A., Litjens G., Menze B., Ronneberger O., Summers R.M. (2022). The Medical Segmentation Decathlon. Nat. Commun..

[16] Dhamdhere K., Sundararajan M., Yan Q. (2018). How Important Is a Neuron?. arXiv.

[17] Ancona M., Ceolini E., Öztireli C., Gross M. (2018). Towards Better Understanding of Gradient-Based Attribution Methods for Deep Neural Networks. arXiv.

[18] Springenberg J.T., Dosovitskiy A., Brox T., Riedmiller M. (2015). Striving for Simplicity: The All Convolutional Net. arXiv.

[19] Gupta M., Mishra A. (2024). A Systematic Review of Deep Learning Based Image Segmentation to Detect Polyp. Artif. Intell. Rev..

[20] Ma J., He Y., Li F., Han L., You C., Wang B. (2024). Segment Anything in Medical Images. Nat. Commun..

[21] Dumitru R.-G., Peteleaza D., Craciun C. (2023). Using DUCK-Net for Polyp Image Segmentation. Sci. Rep..

[22] Vezakis I.A., Georgas K., Fotiadis D., Matsopoulos G.K. EffiSegNet: Gastrointestinal Polyp Segmentation through a Pre-Trained EfficientNet-Based Network with a Simplified Decoder. Proceedings of the 2024 46th Annual International Conference of the IEEE Engineering in Medicine and Biology Society (EMBC).

[23] Lew T., Bonalli R., Pavone M. (2024). Convex Hulls of Reachable Sets. arXiv.

[24] Gamby A.N., Katajainen J. (2018). Convex-Hull Algorithms: Implementation, Testing, and Experimentation. Algorithms.

[25] Sreedhar K., Panlal B. (2012). Enhancement of Images Using Morphological Transformation. Int. J. Comput. Sci. Inf. Technol. (IJCSIT).

[26] Vincent L. (1991). Morphological Transformations of Binary Images with Arbitrary Structuring Elements. Signal Process..

